# Dynamic Modeling of Common Brain Neural Activity in Motor Imagery Tasks

**DOI:** 10.3389/fnins.2020.00714

**Published:** 2020-11-19

**Authors:** Luisa F. Velasquez-Martinez, Frank Zapata-Castano, German Castellanos-Dominguez

**Affiliations:** Signal Processing and Recognition Group, Universidad Nacional de Colombia, Manizales, Colombia

**Keywords:** multi-subject analysis, motor imagery, common spatial patterns, event-related synchronization, functional connectivity

## Abstract

Evaluation of brain dynamics elicited by motor imagery (MI) tasks can contribute to clinical and learning applications. The multi-subject analysis is to make inferences on the group/population level about the properties of MI brain activity. However, intrinsic neurophysiological variability of neural dynamics poses a challenge for devising efficient MI systems. Here, we develop a *time-frequency* model for estimating the spatial relevance of common neural activity across subjects employing an introduced statistical thresholding rule. In deriving multi-subject spatial maps, we present a comparative analysis of three feature extraction methods: *Common Spatial Patterns, Functional Connectivity*, and *Event-Related De/Synchronization*. In terms of interpretability, we evaluate the effectiveness in gathering MI data from collective populations by introducing two assumptions: (i) Non-linear assessment of the similarity between multi-subject data originating the subject-level dynamics; (ii) Assessment of time-varying brain network responses according to the ranking of individual accuracy performed in distinguishing distinct motor imagery tasks (left-hand vs. right-hand). The obtained validation results indicate that the estimated collective dynamics differently reflect the flow of sensorimotor cortex activation, providing new insights into the evolution of MI responses.

## 1. Introduction

Motor imagery (MI) is a dynamic mental state in which an individual performs a mental rehearsal of motor action without any overt output. It is believed that real movements and those performed mentally (imaginary movements) are functionally similar (Stolbkov et al., 2019). Therefore, there is sufficient experimental evidence that MI contributes to substantial improvements in motor learning and performance (Aymeric and Ursula, 2019), games and entertainment, sports training, therapy to induce recovery and neuroplasticity in neurophysical regulation and rehabilitation, and activation of brain neural networks as the basis of motor learning (Machado et al., 2019), and education scenarios (Boe and Kraeutner, 2018; MacIntyre et al., 2018; Suica et al., 2018), where the Media and Information Literacy methodology proposed by UNESCO includes many competencies that are vital for people to be effectively engaged in human development (Frau-Meigs, 2007). These applications reinforce the importance of studying the evolving brain organization to model plastic changes accurately, putting strength on dynamic modeling of temporal, spectral, and spatial features extracted from single channels due to most MI systems rely on them to distinguish distinctive neural activation patterns (Hamedi et al., 2016; Allen et al., 2018).

MI systems handle brain data recorded with electroencephalography (EEG), which is a non-invasive measurement of neural activation and interactions, encoding brain dynamics with high temporal granularity, but at a relatively low spatial resolution (Feng et al., 2019). Integrating spatial filtering techniques can reverse the volume conduction effects to some degree, increasing the EEG spatial resolution. Nevertheless, to enhance the analysis of triggering mental activity, feature extraction approaches are performed to derive distinct EEG spatial maps with varying frequency and time characteristics (Tiwari et al., 2018). To begin with, Filter-Bank Common Spatial Patterns are a popular algorithm in MI systems that discriminate multichannel EEG signals by highlighting differences while minimizing similarities, selecting frequency bands appropriately (Baig et al., 2019). Also, Functional Connectivity (FC) networks are extracted because a better understanding of MI mechanisms requires knowledge of the way the co-activated brain regions interact with each other (Stavrinou et al., 2007). Accordingly, the *wPLI* metric of EEG functional connectivity can account for linear brain interactions but is also expected to be sensitive to non-linear couplings Imperatori et al. (2019). Another approach for characterizing the imaged hand movements is to quantify frequency alterations in time-varying responses to a stimulus (event) through the so-termed Event-Related De/Synchronization (ERD/S), presenting a significant correlate of localized cortical oscillatory activity (Juan et al., 2019). When imagining one hand moving, an increase/decrease in the power of μ and β rhythms becomes more potent in the sensorimotor (electrodes C3 and C4) and pre-motor (Cz) areas located contralaterally to the hand involved in the task (Wierzgała et al., 2018). Due to the non-stationarity of EEG data, however, the effectiveness of feature extraction procedures is reduced in deriving distinct EEG spatio-spectral patterns. Several factors can affect, among others, the following: movement artifacts during recording, temporal stability of mirroring activation over several sessions differs notably between MI time intervals (Friedrich et al., 2013; Pattnaik and Sarraf, 2018), low EEG signal-to-noise ratio, poor performance in small-sample settings (Park and Chung, 2019), and inter-subject variability in EEG dynamics (Saha et al., 2018). Along with variability in the signal acquisition, another circumstance that leads to low accuracy scores is that some subjects may have brain networks, not sufficiently developed for practicing MI tasks (Ahn and Jun, 2015). As a result, the performance of MI systems varies considerably across and within-subjects, severely degrading their reliability.

To compensate for the variability of EEG dynamics, novel approaches are being developed to integrate information across subjects within a collective framework, combining individual feature sets of neural dynamics to improve the brain representation robustness, as explained in Bigdely-Shamlo et al. (2018). Thus, under the assumption that temporal signatures from an evoked neural activity are similar across subjects, group models can be extracted for decoding the multi-subject mental responses to complex stimuli without explicitly representing the elicitation (Fazli et al., 2015). Several strategies for raw data aggregation can be implemented for building group inferences, including serial/parallel combinations of subject-level feature sets to form a more extensive multi-subject array (Lio and Boulinguez, 2016). Instead, data-driven approaches have also been employed to infer collective feature structures, like joint diagonalization (Gong et al., 2018), temporally constrained sparse representation (Zhang et al., 2019), canonical correlation analysis (de Cheveigné et al., 2019), and versions derived from independent Component Analysis (Emge et al., 2018; Huster and Raud, 2018; Bhinge et al., 2019), among others.

For interpretation purposes, the topographic representation is commonly computed to display the spatial distribution of the extracted common neural dynamics. Nonetheless, the building of multi-subject models implies the accurate aggregation of time-frequency patterns extracted from EEG dynamics across the subjects by adequately selecting the domain parameters (i.e., time window length and filter bandwidth setup) (Huster and Raud, 2018). Moreover, the aggregation can face a different dimensionality derived from the feature extraction methods involved. Due to the difference in captured dynamics, each engaged extraction method differently reflects the flow of sensorimotor, being one of the issues that arise in identifying group relationships confidently (Bridwell et al., 2018). Besides, to evaluate computational network models, there is a need to establish the meaning of the aggregation of extracted brain-activity patterns (Kriegeskorte et al., 2008). Hence, another issue to consider is to assess the ability of multi-subject sets to preserve the main properties (i.e., the spatial distribution of brain neural activity throughout time and spectral domains) extracted from single-subject models.

Here, we develop a dynamic model for estimating the common neural activity across subjects to provide new insights into the evolution of collective mental imagery processes. After the preprocessing stage, the *t-f* EEG signal set is fed into a feature extraction algorithm to improve the efficiency of triggering activity representation. Then, we employ a statistical thresholding algorithm to extract a multi-subject model that provides a set of confident estimates contributing the most to discriminating between MI tasks. We present a comparative analysis of the feasibility of three popular feature extraction methods in deriving multi-subject spatial maps: *Common Spatial Patterns, Functional Connectivity*, and *event-related de/synchronization*. The obtained validation results indicate that the estimated collective dynamics reflect the flow in the sensorimotor cortex activation differently. Therefore, the common model addresses inter-subject and inter-trial variability sources individually, depending on the engaged extraction method.

The paper is organized as follows: section 2 describes the validated database and methods that are carried out; section 3 presents the experimental setup as well as the performed outcomes; section 4 introduces a detailed discussion of the attained results, providing the main conclusions of this work.

## 2. Materials and Methods

### 2.1. Description Tested of Bi-task MI Databases

#### 2.1.1. Dataset D-I

We perform experimental validation in nine subjects (*M* = 9) of Dataset 2a^1^, holding EEG signals acquired from the scalp by a *C*-channel montage (*C* = 22). Every raw EEG channel ***x***(*c*)∈ℝ^*T*^ was sampled at 250 *Hz* (i.e., at the sample rate Δ*t* = 0.004 *s*). To perform each MI task (left and right hand with labels noted as λ∈{*l, l*′}, respectively), a short beep noticed the trial beginning, followed by a fixation cross that appeared on the black screen within the first 2 *s*-interval. An arrow (cue) appeared during 1.25 *s*, and pointed to the induced direction. Then, each subject performed a demanded MI task while the cross reappeared within the next time interval, starting from 3.25 *s* to the recording end. All signals were collected in six runs separated by short breaks, performing *N*_λ_ = 72 trials per class and each lasting *T* = 7 *s*. Of note, we only examined the labeled trials for which artifact removal had been applied.

#### 2.1.2. Dataset D-II

**Figure 1:**
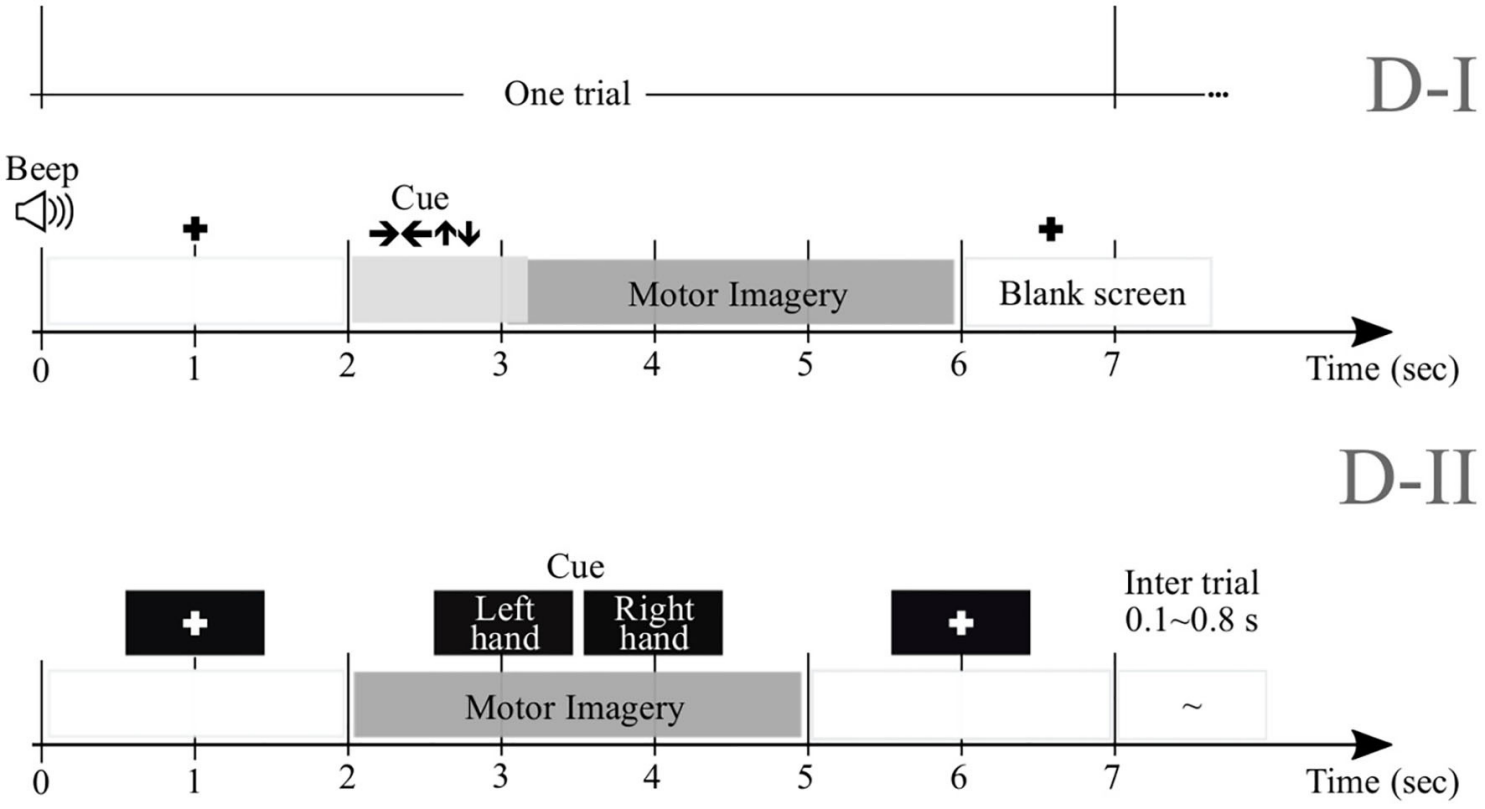
Trial timing used to implement the MI paradigm of the tested databases.

We also examine this collection that holds EEG data obtained from fifty-two subjects (although only *M* = 50 are available) using a 10−−10 placement electrode system with *C* = 64 channels^2^. Every channel ***x***(*c*) lasted *T* = 7 *s* and sampled at *F*_*s*_ = 512 *Hz*. At the trial beginning, a fixation cross was presented on a black screen within a period that lasted 2 *s*. Then, a cue instruction (related to either label—*l* or *l*′) appeared randomly on the screen for 3 *s* that inquired each subject to imagine moving his/her fingers, starting to form the index finger and proceeding to the little finger and touching each to their thumb. Afterward, a blank screen was shown at the beginning of a break period, lasting randomly between 4.1 and 4.8 *s*. For completing a single run, this procedure was repeated over 20 times and stopped at the end to fulfill a written cognitive questionnaire (Cho et al., 2017). Every subject performed five or six runs. Figure 1 displays the trial timing used to implement the MI paradigm of the tested databases: D-I and D-II.

### 2.2. Subject-Level Extraction of *t-f* Feature Dynamics

Using a sliding window approach, the short-time feature set is extracted from multiple frequency bands to build the labeled subject-level model of feature dynamics. At this stage, we compare the following methods of *t-f* feature extraction: *Common Spatial Patterns, Functional Connectivity*, and *Event-Related De/Synchronization*.

#### 2.2.1. Estimation of Common Spatial Patterns

Given a filter-band-passed trial matrix $X_{nf\tau }^{\lambda }\in {\mathbb{R}}^{C\times T},$ *n*∈*N*_λ_, *f*∈*N*_*f*_, CSP finds within the time-windowed partition τ∈*N*_τ_ the linear transformation vector $w_{f\tau }\in {\mathbb{R}}^{C}$ that maximizes the Rayleigh Quotient (RQ) between both labels λ, defined as follows (Aghaei et al., 2016):

(1) $$\underset{\forall w_{f\tau }}{\max}J=\frac{w_{f\tau }^{⊤}\Sigma_{f\tau }^{l}w_{f\tau }}{w_{f\tau }^{⊤}\Sigma_{f\tau }^{l}+\Sigma_{f\tau }^{l^{\prime }}w_{f\tau }},\text{ s}.\text{t}.:\;\|w{\|}_{2}=1$$

where the matrix $\Sigma_{f\tau }^{\lambda }\;=\;E\left\{X_{nf\tau }^{\lambda }X_{nf\tau }^{\lambda ⊤}:\forall n\in N_{\lambda }\right\}$ is the simplest estimate of the class data variance, computed at a frequency *f* and sliding window τ. The notations ||·||_*p*_ and *E*{·:∀*n*} stand for ℓ_*p*_-norm and expectation operator across a variable *n*, respectively. Then, the sampled EEG data $X_{nf\tau }^{\lambda }$ are filtered through the learned spatial matrix $W_{f\tau }\in {\mathbb{R}}^{\hat{K}\times C}$, holding $\hat{K}\leq C$ transformation components. Further, the projected data $Z_{nf\tau }^{\lambda }\;=\;W_{f\tau }X_{nf\tau }^{\lambda }$ are obtained using only $\hat{K}\;=\;2k$ representative terms (namely, *k* first and *k* last rows), from which the feature vector $d_{nf\tau }\in {\mathbb{R}}^{2k}$ is then extracted as below (Brandl et al., 2016):

(2) $$d_{nf\tau }=\log\;(diag(\mathrm{var}\left\{Z_{nf\tau }^{\lambda }\right\})),\;d_{nf\tau }\subset D\in {\mathbb{R}}^{(N_{l}+N_{l^{\prime }})\times Q}$$

where *var*{·} denotes the variance operator. Note that the obtained feature matrix ***D*** = [***d***_*nfτ*_:*n*∈*N*_λ_] holds *Q* = *N*_*f*_×*N*_τ_×2*k* concatenated features, which are extracted from each MI recording trial.

Relying upon the inverse transformation matrix $W_{f\tau }^{-}$, ultimately, we model the CSP-based dynamics of the spatial *t*−−*f* patterns of brain activation, which are computed as below:

(3) $$\theta_{J}(f,\tau )=vec{\left\{W_{f\tau }^{-}\right\}}^{⊤}$$

where the vector $\theta_{J}\left(f,\tau \right)\in {\mathbb{R}}^{C}$ gathers the *t-f* contribution from *c*-th EEG channel in terms of distinguishing between both labels, being learned over the whole trial set and calculated by the highest variance value (i.e., $\hat{K}\;=\;1$).

#### 2.2.2. Computation of Functional Connectivity of Brain Networks

To investigate the pairwise inter-channel relationship, we use the *weighted Phase Locking Index* (*wPLI*) as an FC metric that quantifies the asymmetry of the phase difference distribution between two specific channels *c, c*′ (with ∀*c, c*′∈*C, c*≠*c*′), being each one estimated across the trial set, ∀*n*∈*N*_λ_, as follows (Bastos and Schoffelen, 2016):

(4) $$\begin{array}{l} \phi_{cc^{\prime }}(f,\tau |\lambda )\\ =\frac{\left|\text{E}\left\{|\Delta \Phi_{cc^{\prime }}^{\left(n\right)}(f,\tau ;c,c^{\prime }|\lambda )|\;\mathrm{sgn}\;(\Delta \Phi_{cc^{\prime }}^{\left(n\right)}(f,\tau ;c,c^{\prime }|\lambda )):\forall n\right\}\right|}{\text{E}\left\{|\Delta \Phi_{cc^{\prime }}^{\left(n\right)}(f,\tau ;c,c^{\prime }|\lambda )|:\forall n\right\}}, \end{array}$$

where notation sgn stands for *sign* function and $\Delta \Phi_{c,c^{\prime }}^{\left(n\right)}\left(;|\right)\in \mathbb{R}\left[0,\pi \right]$ is the instantaneous phase difference computed through the continuous wavelet transform coefficients $W_{cc^{\prime }}^{\left(n\right)}\left(f,\tau ;c,c^{\prime }|\lambda \right)\in {\mathbb{R}}^{+}$ by the ratio $\Delta \Phi_{cc^{\prime }}^{\left(n\right)}\left(;|\right)\;=\;W_{c}^{\left(n\right)}\left(;|\right)W_{c^{\prime }}^{\left(n\right)}\left(;|\right)/|W_{c}^{\left(n\right)}\left(;|\right)||W_{c^{\prime }}^{\left(n\right)}\left(;|\right)|$.

The *wPLI* metric, $\phi_{cc^{\prime }}\left(f,\tau |\lambda \right)\in {\mathbb{R}}^{v}$, is normalized to highlight the connectivity patterns generated by each evoked task, being each mean-value averaged over the trial set within a given baseline interval Δ*T*_0_. Thus, we obtain the inter-channel connectivity vector through the following marginal across the node set: $\hat{\phi }\left(f,\tau |\lambda \right)=\underset{v\in V}{\sum }\phi \left(f,\tau ;v|\lambda \right)$ and the pairwise variable *v*∈{*c, c*′∈*V, c*≠*c*′}, where *V* = *C*(*C*−−1)/2 is the number of considered paired links. Therefore, we model the dynamics extracted from $\hat{\phi }\left(,|\right)\in {\mathbb{R}}^{C}$ according to the following rule:

(5) $$\theta_{\hat{\phi }}(f,\tau |\lambda )=[\hat{\phi }(f,\tau ;c|\lambda ):c\in C]$$

#### 2.2.3. Assessment of Event-Related (De-)Synchronization

This time-locked change of ongoing EEG is a somatotopical organized control mechanism that can be generated intentionally by mental imagery and has specific frequency-band interpretation. Using each *c*-th measured EEG recording $x_{nf}^{\lambda }\left(c\right)$, the ERD/S estimation is performed, at a frequency band *f* and sample τ, by squaring of samples and averaging over EEG trials to compute the variational percentage (decrease or increase) in the EEG signal power regarding a reference interval as follows (Dai and Wei, 2017):

(6) $$\zeta (f,\tau ;c|\lambda )=(\xi (f,\tau ;c|\lambda )-\bar{\xi }(f;c|\lambda ))/\bar{\xi }(f;c|\lambda )$$

where $\xi \left(f,\tau ;c|\lambda \right)=E\left\{|x_{\tau }^{\lambda }\left(c\right){|}_{nf}^{2}\in x_{nf}^{\lambda }\left(c\right):\forall n\right\}$ is the power scatter averaged across the trial set and $\bar{\xi }\left(f;c|\lambda \right)=E\left\{\xi \left(f,\tau ;c|\lambda \right):\forall \tau \in \Delta T_{0}\right\},$ with $\bar{\xi }\left(f;c|\lambda \right)\in \mathbb{R}$, is the trial power scatter averaged over the reference time interval τ_0_⊂*T*, being *T*∈ℝ^+^ the recording time span.

Given a label λ, therefore, we represent its corresponding ERD/S-based dynamics by computing the functional in Equation. (6) across all channels, that is:

(7) $$\theta_{\zeta }(f,\tau |\lambda )=[\zeta (f,\tau ;c|\lambda )\in \mathbb{R}:\forall c\in C],\;\theta_{\zeta }(;|)\in {\mathbb{R}}^{C}$$

As a result, we estimate the subject-level model of *t-f* feature dynamics $\left\{\theta_{\eta }^{\left(m\right)}\left(f,\tau |\lambda \right):\forall f,\forall f\tau \right\}$ extracted by each method (noted by η = {*J*, ζ, ϕ}) for *m*-th individual. The model contains the electrode set contribution, $\theta_{\eta }^{\left(m\right)}\left(,|\right)\in {\mathbb{R}}^{C}\left[0,1\right]$, estimated at frequency *f*, time τ, and given a label λ (besides the CSP-based spatial filtering that resumes in a single model the joint influence of both labels).

### 2.3. Group-Level Extraction of Multi-Subject *t-f* Dynamics

The goal is to capture the inter-subject *t-f* feature dynamics, which are to be considered as prevalent in the group/population level, guaranteeing that the MI responses are measured from subjects under the equivalent conditions of the experimental paradigm. We assume that the data collected are statistically independent between individuals. Under this assumption, the common assessments of the extracted feature sets become confident as they are present in a higher number of subjects. In this regard, the subject-level model provides a set of confident estimates that contributes the most to discriminating between tasks using the following supervised, statistical thresholding algorithm (Padilla-Buritica et al., 2020):

$$\kappa_{m}^{f}(c)=\{\begin{array}{ll} 1, & ℳ\{\theta^{cfm}(\tau )|\lambda :\forall \Delta_{T_{i}}\}<p\\ 0, & \text{Otherwise}, \end{array}$$

where the rule $M\left\{\cdot |\lambda :\forall \Delta_{T_{i}}\right\}$ computes the statistical discrepancy/consitency along Δ_*T*_*i*__ time window using a non-parametric Mann-Whitney test under the null hypothesis that the distribution of all channels is equal. Thus, $\kappa_{m}^{f}\in^{C}$ holds the *p*-values for all considered channels. Besides, we apply the Kolmogorov-Smirnov and Bartlett's tests to address these issues since the estimated set can present failures related to normality and homoscedasticity. Finally, because we know the channel's discriminant capacity of each subject, and assuming the independence of the performed validation, we accomplish a group-level analysis using the positive False Discovery Rate as a robust statistical correction in the multiple-subject comparison testing.

We also evaluate the ability of multi-subject sets to preserve the main properties obtained from the single-subject model sets. Namely, we quantify the variations in the spatial distribution of common brain neural activity raised by the heterogeneity between subjects due to the reported dependence of individual skills for adequately practicing the MI tasks. As mentioned before, we build a group-level model for BCI-literacy group in either tested database. Thus, we appraise the inter-group topographical variability between attained multi-subject dynamics. Besides, the intra-similarity of the extracted individual dynamics is presented to estimate the influence of each subject on the performed dynamic multi-subject model. So, we compute multi-subject models over all subjects. Then, the multi-subject model is computed by removing the subject having the worst accuracy. Next, the multi-subject model is evaluated by removing the tow worst subjects, and so on, desegregating each individual by the ranked accuracy.

Here, we calculate the topographical similarity using a generalized inner product measured between a couple of spatial dynamics, **η** and **η′**, projecting the difference of data onto a reproducing kernel Hilbert space through a Gaussian kernel as follows (Mikalsen et al., 2018):

(8) $${\langle \eta ,\eta^{\prime }\rangle }_{\sigma }=\exp\;\left(-\|\eta -\eta^{\prime }{\|}^{2}/\sigma^{2}\right)$$

where σ∈ℝ^+^ is a ruling parameter for which the estimate is obtained from the MI segment.

## 3. Experimental Set-Up

**Figure 2:**
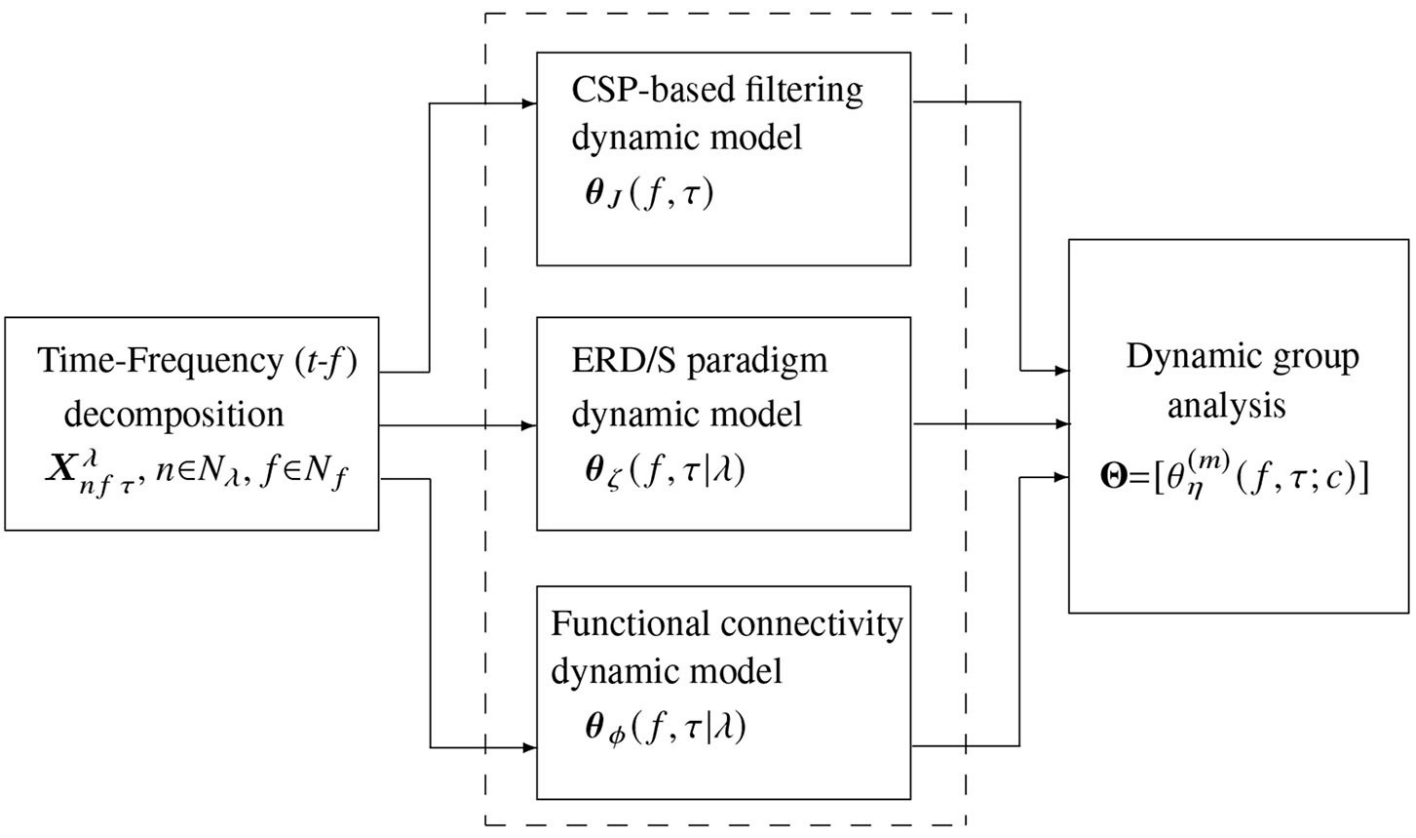
Scheme illustrating the stages of common dynamic modeling proposed for brain neural activity in motor imagery tasks. The evaluated *t-f* feature extraction methods are contained in the dashed box.

Validation of the proposed approach for common-dynamics modeling of brain neural activity comprises three stages (see the pipeline in Figure 2): (i) Decomposition of input EEG time-series into a frequency-specific temporal representation; (ii) Subject-level feature extraction of *t-f* dynamics, encoding the electrode contribution, (iii) Extraction of multi-subject *t-f* dynamics. We performed the pairwise distance between individuals on the subject-level and group-level stages during the evaluation. Also, visual inspection of the obtained extracted *t-f* dynamics is presented, stressing on the physiological interpretability of results.

### 3.1. Pre-processing of EEG Signals

Initially, every raw EEG channel ***x***(*c*) is band-pass filtered in the frequency range *f*∈[4−−40] *Hz* using a filterbank of *N*_*f*_ = 17 filters with 2 *Hz* bandwidth overlap. For either considered database, the bandwidths are selected as to cover μ and β, widely reported for practicing MI tasks (Dai and Wei, 2017). However, as suggested in Graimann et al. (2002), we split β oscillation into three bandwidths, namely, [16−−20], [20−−24], and [24−−28] *Hz*. Spectral partitioning is carried out within the following time-window lengths (namely, τ_*J*_ = [0.5, 1, 1.5, 2] *s* with 90% overlapping). Then, to provide physiological interpretation according to the implemented experimental paradigm of MI, the dynamics are analyzed at the following representative intervals of interest: Δ*T*_1_ = [0−−2] *s* (interval prior to cue-onset or task-negative state), Δ*T*_2_ = [0.8−−2] *s* (cue-onset interval), Δ*T*_3_ = [2.6−−4.6] *s* (motor imagery interval), Δ*T*_4_ = [3.8−−5.8] *s* (decaying motor imagery interval), and Δ*T*_5_ = [4.4−−6.4] *s* (break period).

For addressing the volume conduction problem, all *t-f* patterns are computed by performing the Laplacian filter previously over the input EGG data to improve the spatial resolution of EEG recordings, avoiding the influence of noise coming from neighboring channels (Carmen et al., 2011). We implemented the spatial filtering using *Biosig Toolbox*^3^.

One aspect significantly influencing the extraction of dynamics is the subject's ability to evoke high-quality and recognizable MI responses. To manage inter-subject variability, we assume the rationale by which the more developed the individual brain network, the higher the accuracy in distinguishing between MI tasks. Accordingly, depending on whether a subject has skills to master MI applications, both databases are split into two subject assemblies: BCI-literacy and BCI-illiteracy.

### 3.2. Single-Subject Dynamics Performed by Common Spatial Patterns

After selecting the bandwidth setup, the starting point to implement the short-time feature extraction of *t-f* CSP dynamics is the computation of RQ time-series by adequately tuning the time window length τ_*J*_ and by fixing the variance of the surrogate space to the first eigenvectors (*k* = 3) of the matrix ***W***_*fτ*_. Therefore, using a tailored software, we extract two feature sets from each time-frequency segment: *D*∈ℝ^144 × 102^ for D-I and *D*∈ℝ^200 × 102^ for D-II.

**Figure 3:**
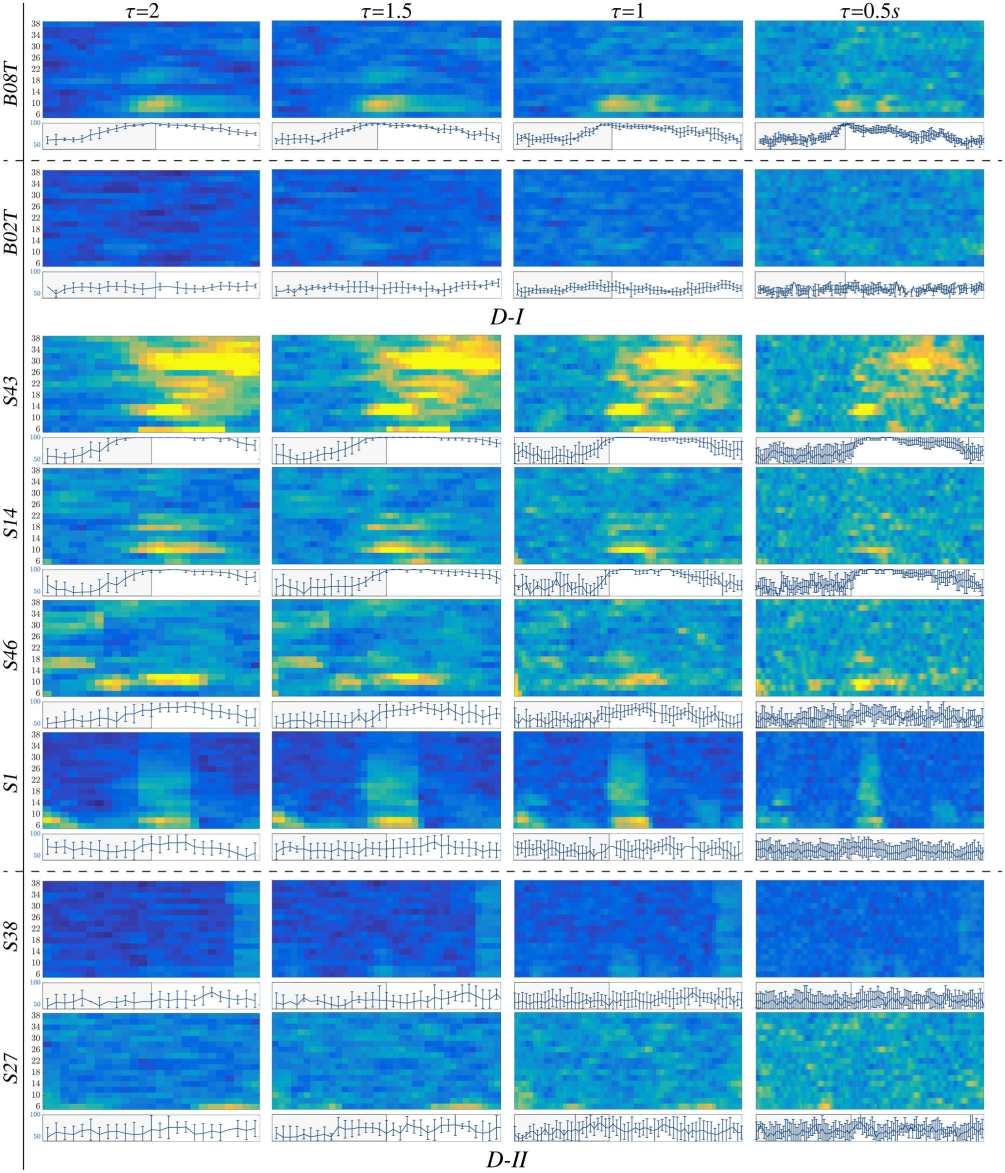
*t-f* CSP dynamics computed for representative individuals (BCI-literate and BCI-illiterate) belonging to each tested dataset (D-I and D-II). Under each plot, the accuracy evolution over the interval of neural activation *T* is displayed for a fixed value of window τ_*J*_.

For illustration purposes, we present the extracted CSP dynamics just for several representative subjects, who have been reported as having high accuracy (*BO8T* for database D-I, and *S43, S14, S46* for D-II) and low accuracy (*BO2T* for D-I, and *S10, S38*, and *S2* for D-II). Figure 3 presents the *t-f* features performed individually, revealing a very changing behavior of the assessments. This fact becomes evident in the accuracy evolution over time displayed under each plot of CSP-based dynamics. The time-evolving accuracy reveals that the optimal value of τ_*J*_ provides the best accuracy and varies widely across subjects and ranges within the entire span of the tested window length. Table 1 presents the mean and standard deviation of accuracy, averaged across the subject set, indicating that the average performance tends to increase as the length τ_*J*_ shortens. However, the accuracy degrades for the smallest window. Because one more concern in choosing τ_*J*_ is the need for sufficient statistics to estimate the collective dynamics, we fix the optimal window to $\tau_{J}^{*}\;=\;1$ *s* as a tradeoff between accuracy and an adequate number of samples on the interval of interest in implementing the multi-subject modeling.

**Table 1:** Accuracy of the extracted RQ time-series, varying τ_*J*_.

	**D-I**			**D-II**		
**τ_*J*_**	**All**	**Literate**	**Illiterate**	**All**	**Literate**	**Illiterate**
0.5	83.8 ± 6.6	91.3 ± 5.3	80.5 ± 4.0	85.6 ± 9.0	92.1 ± 7.6	77.6 ± 3.2
1.0	**84.1 ± 7.9**	94.1 ± 4.1	79.8 ± 4.5	**85.7 ± 10.9**	94.1 ± 5.5	75.2 ± 7.5
1.5	82.8 ± 9.8	93.0 ± 5.4	78.0 ± 7.1	87.6 ± 11.3	96.0 ± 4.0	77.2 ± 10.0
2.0	82.1 ± 11.3	95.0 ± 5.3	76.5 ± 8.3	87.2 ± 11.6	95.4 ± 3.9	77.0 ± 11.8

*All stands for averaging across the while group, while Literate and Illiterate represent the corresponding subject subset computation*.

The bold values indicate the accuracy performed by the optimal window fixed to $\tau_{J}^{*}=1$ s.

To manage the significant impact of inter-subject variability on the reached accuracy, we employ a neurophysiological predictor of BCI performance to divide the evaluated subjects into two clustered assemblies: BCI-literacy, or users with the ability to produce reliable and reasonably robust differences in neural activity between distinct MI tasks (e.g., left-hand vs. right-hand) (Allison and Neuper, 2010), and BCI-illiteracy, or individuals who are not accurate enough to control the MI application.

**Figure 4:**
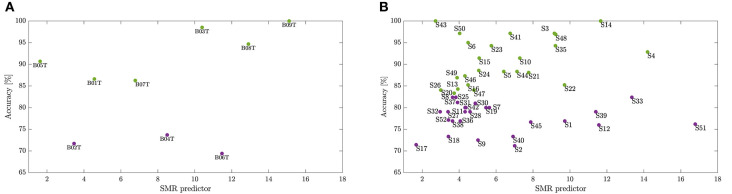
Division into BCI-literacy and BCI-illiteracy. Scatter plots performed by the neurophysiological predictor for each database, D-I **(A)** and D-II **(B)**.

**Figure 5:**
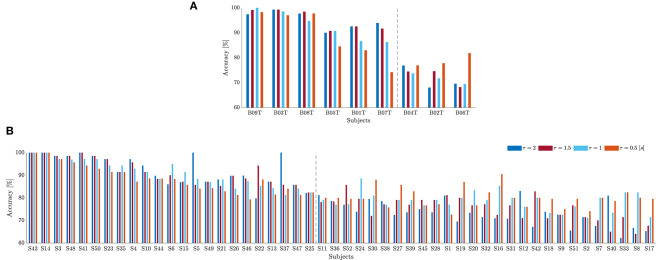
Individual classifier accuracy of MI tasks estimated for D-I **(A)** and D-II **(B)** at different window lengths (Subjects are ranked in decreasing order of performance at $\tau_{J}^{*}\;=\;1$). The dashed line separates the BCI-literate subjects from BCI-illiterate ones.

As suggested in Blankertz et al. (2010), we cluster the whole subject set into two mutually exclusive assemblies by removing points of the sample that have the 10% largest Malahanobis distance to the data center. For each tested database, Figure 4 presents the obtained scatter plots using the neurophysiological predictor, having as input data the average values of mean and standard deviation computed in each case of τ_*J*_. To clarify the clustering results, we rank the subject set in decreasing order of the average discrimination accuracy in Figures 5A,B (D-I,D-II), where a dashed vertical line separates both assessed assemblies, displaying the individual performance estimated at different window lengths. As a result, the D-I collection holds five BCI-literacy subjects and four BCI-illiterate. In turn, D-II contains 15 BCI-literacy subjects, and the remaining 35 are BCI-illiterate.

Also, we appraise the spectral contribution by the marginal values of CSP dynamics on each bandwidth of *f*, as seen on the plots depicted in Figure 3. Thus, the individuals with high accuracy (yellow spots) have a few spectral components powerfully localized, showing that the more contributing waveforms are μ and β. In contrast, the low-accuracy subjects have a weak contribution that tends to spread over all bandwidths, increasing the variability of estimated CSP patterns.

Another aspect to consider is the representative intervals of interest that influence the most in the MI responses. Thus, in the cases of individuals with higher performance (BCI-literacy), the best accuracy is estimated within Δ*T*_3_, when the most increased neural activation is expected to take place according to the used trial timing. By the opposite, subjects with lower accuracy (BCI-illiteracy) deliver better estimates of performance outside the MI period Δ*T*_3_; their high irregularity may explain this incorrect time localization of relevant MI responses in following the experimental paradigm (Brockmeier, 2014). Consequently, the more scattered over time and frequency domains the extracted CSP patterns, the lower the accuracy achieved by the subjects.

From Figure 3, it may be concluded that every subject rules the RQ evolution through τ separately. This restriction poses a challenge for extracting multi-subject dynamics, for which a unique value of time window must be determined across the whole subject set. Another critical point hindering the estimation of RQ maps is the use of CSP-based filtering that demands a long window τ, decreasing the accuracy of the performed *t-f* feature dynamics so that the variability of inter-subject dynamics increases notably due to inherent non-stationarity, artifacts, a low signal-to-noise ratio of EEG signals, individual differences in cortical activity resulting in variations covariance matrix and consequently estimated spatial filters (Wang and Zheng, 2008).

**Figure 6:**
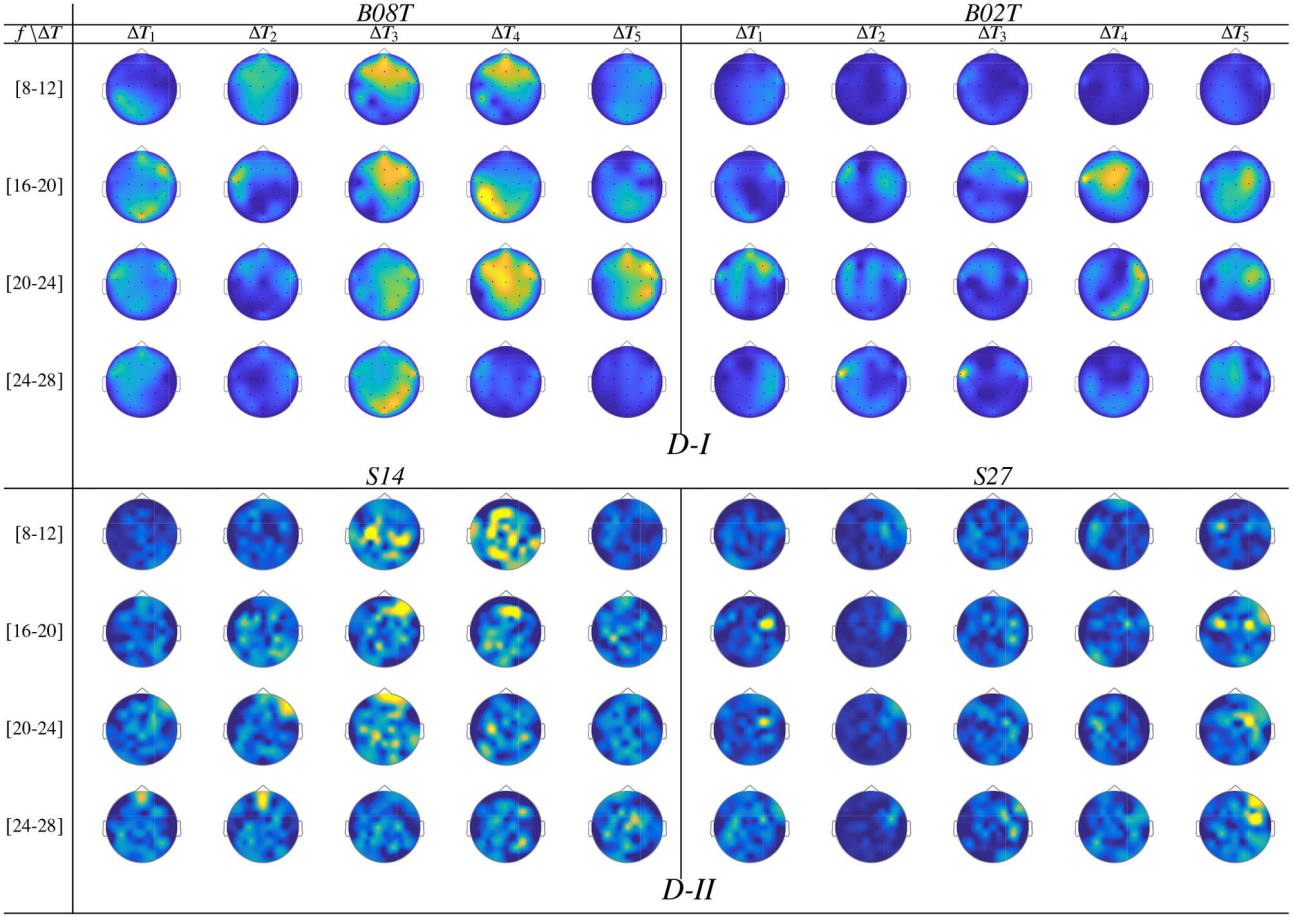
Topographical arrangement presenting the *t-f* dynamics of CSP patterns **θ**_*J*_(*f*, τ) for the subjects performing the best and worst accuracy of each validated database.

Once the domain parameters (i.e., time window length and filter bandwidth setup) are selected, we compute the topographical representation of brain neural dynamics **θ**_*J*_(*f*, τ) performed by CSP. For the sake of illustration, Figure 6 displays the neural dynamics performed by representative subjects of both data subsets, that is, *BO8T* and *BO2T* in D-I, and *S14* and *S27* for Dataset D-II. As seen, the filter-bank bandwidths of BCI-literacy individuals that contribute the most fall into μ and β oscillations, involving activity in the centro-lateral primary motor area, supplementary motor area, and primary somatosensory area, as reported in Catharina et al. (2015). On the contrary, the illiterate subjects *BO2T* and *S27* hold the spectral contribution that is more localized over the pre-frontal to the mid-central area, being lower on μ and spreading extensively, but with a much lower contribution. Overall, the neural activation dynamics **θ**_*J*_(*f*, τ) are mostly confined within the cue-onset and MI intervals but rising distinctly in the latter MI period of either subject. Note a few spurious activities within Δ*T*_1_, which may be caused by the overlapping window of estimation.

### 3.3. Single-Subject Dynamics Extracted by Functional Connectivity

Before extracting the *t-f* functional connectivity features, we perform the preprocessing stage of Laplacian filtering, fixing channel Cz as reference (Daly et al., 2012). Nevertheless, the influential non-stationarity nature of EGG data rules a high variability between trial sets, fluctuating on multiple time-scales that range from milliseconds to seconds (Lang et al., 2012). To meet this condition, the estimator in Equation (4) is performed by adjusting the short-time window to a small length, τ_ζ_ = 0.1 *s* as presented in Padilla-Buritica et al. (2019). Of note, all connectivity assessments are computed using the FielTrip toolbox (Oostenveld et al., 2011).

**Figure 7:**
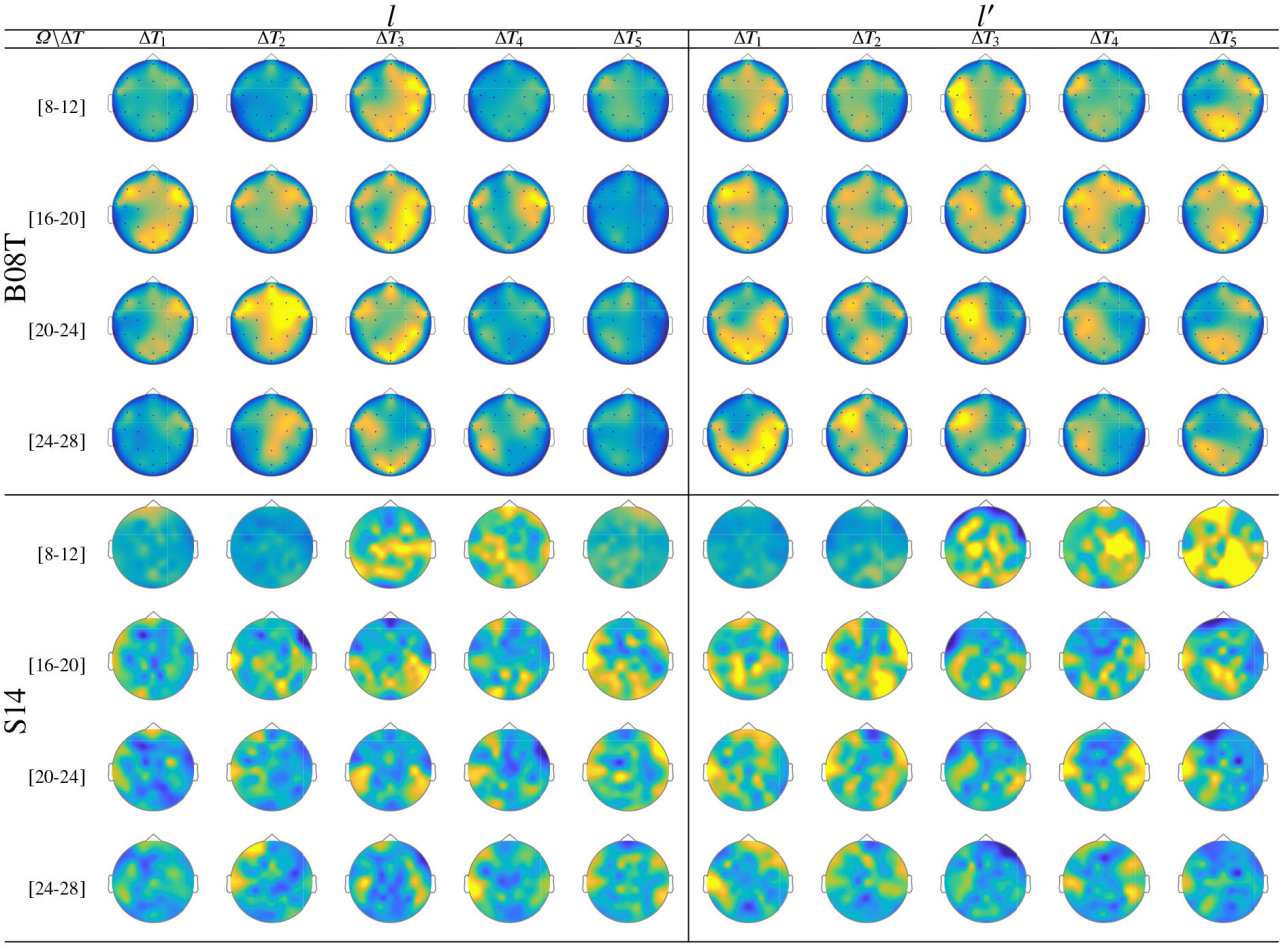
Labeled brain neural dynamics, **θ**_ϕ_(*f*, τ|λ), extracted by the functional connectivity method using *wPLI* and performed by the representative literate individuals: *B08T* (upper plots) and *S14* (lower plots).

Figure 7 displays the dynamic of *t-f* features extracted from *B08T* (upper plots) and *S14* (lower plots) that explain a high inter-subject variance of the performed FC patterns. Also, a considerable number of acting nodes is achieved by either individual within the segment before onset, Δ*T*_1_. This background FC activity has been previously associated with some resting-state networks (overlapping the primary motor, visual and auditory networks, the default mode network, and higher-order attention networks), which are distributed over the frontal, central, parietal, and occipital areas (Van Den Heuvel and Pol, 2010). The FC activity presents a similar behavior over the neighboring interval (Δ*T*_3_ and Δ*T*_4_), including the ending of each MI task and the break period.

In the representative MI interval, Δ*T*_3_, the FC patterns performed by *B08T* and *S14* differ between both classes, covering multiple cortical regions. Thus, neural connectivity is more powerful over the corresponding contralateral hemisphere associated with the parameter-parietal network, as detailed in Hanakawa et al. (2008). Specifically, as stated in Kasahara et al. (2015), the Supplementary motor area, the Pre-motor cortex, and the posterior parietal cortex are interconnected. An additional aspect to highlight is the evenness of FC dynamics performed by individuals belonging to DB-I due to the lower number of electrodes, yielding lower resolution than the one assessed in DB-II.

### 3.4. Single-Subject Dynamics Extracted by Event-Related De/Synchronization

**Figure 8:**
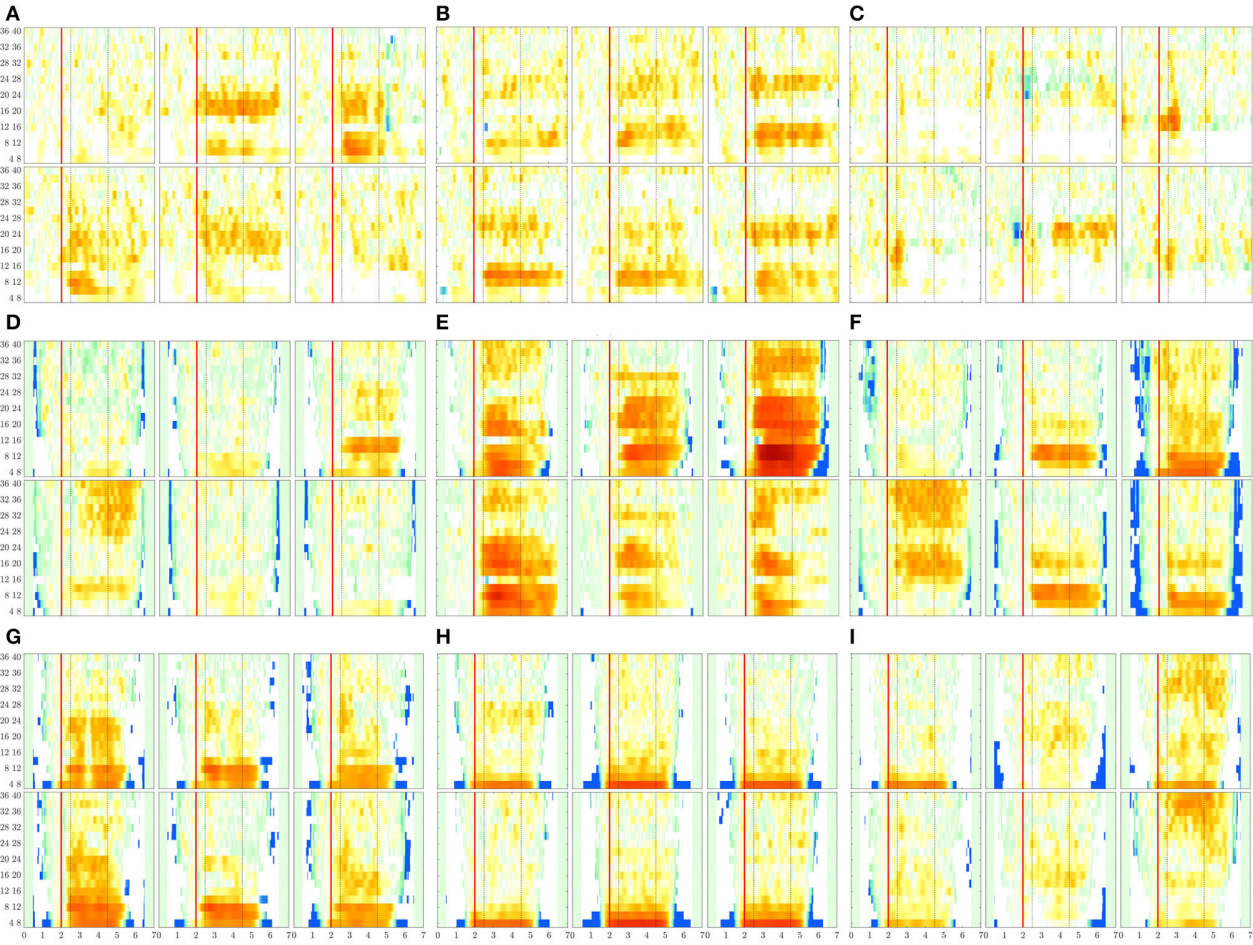
ERDs maps of channels placed above the sensorimotor cortex areas (C3, Cz, and C4) performed by each subject. The rhythm modulation amplitudes are presented for label *l*—left hand (upper row) and *l*′—right hand (bottom row). **(A)** *B08T*, **(B)** *B01T*, **(C)** *B02T*, **(D)** *S43*, **(E)** *S14*, **(F)** *S46*, **(G)** *S01*, **(H)** *S38*, and **(I)** *S27*.

Further, we extract the ERD/S dynamics from the filtered trial matrix $X_{N_{f}N_{\tau }}^{\lambda }$ by fixing the following parameter values: τ_ζ_ = 0.004*s* (i.e., time window equals the sample rate), the reference interval Δ*T*_0_ = 0.5−−1.5 *s*, and the significance value is chosen as 1% in *z*-score approach (see Equation 6), as suggested by Scherer and Vidaurre (2018). Figure 8 presents the *t-f* patterns of ERD modulation performed individually, marking with a red line the cue onset time at 2 *s*, and with gray dotted line the MI segment, Δ*T*_3_.

The rhythm modulation of ERD/S patterns allows interpreting the experimental paradigm of MI tasks, as seen in Figure 8 that displays the representative changes of *t-f* patterns estimated for several representative individuals. In the case of literate subjects (*B08T, B01T, S43, S14*, and *S46*), the modulation amplitudes are placed over the sensorimotor cortex area. That is, the contralateral electrode power (i.e., electrode C3 for right-hand and C4—left-hand) decreases step-wise, just before the task onset (marked with a red line), and then gradually increases after the corresponding task ends. This behavior holds within the MI interval and is significant in [8−−12] and [16−−24] Hz bandwidths. Nevertheless, the synchronization patterns are different from each other regardless of their achieved very close accuracy, confirming the widely reported inter-subject variability in practicing MI tasks (Samek et al., 2012).

For the illiterate subset (*B02T, S01, S38, S27*), the ERD/S dynamics have weak amplitudes clustered in irregular shape patterns so that the difference in time-locked responses between contralateral and ipsilateral tends to disappear, as it is the case for *B06T* and *B02T* for which the neural synchronization effect can be barely observed because of their high inter-subject variability.

Another result to point out is the variational increase in the ERD modulation perceived on either electrode of sensorimotor cortical areas. As observed in Figure 8, a robust right-hand modulation appears at the contralateral C3 electrode in most of the individuals. In fact, the higher the accuracy, the more intense the modulation amplitudes. This effect may be linked to left hemisphere dominance, which is commonly reported for motor sequencing tasks (Haaland et al., 2004). Alternatively, the left-hand modulation located at C4 is less evident at μ and β bands, appearing in *S38* and *S27*. Furthermore, in some cases, the modulation is also presented at the ipsilateral C3 electrode, lessening the ERD/S mechanism, and probably leading to poor accuracy.

**Figure 9:**
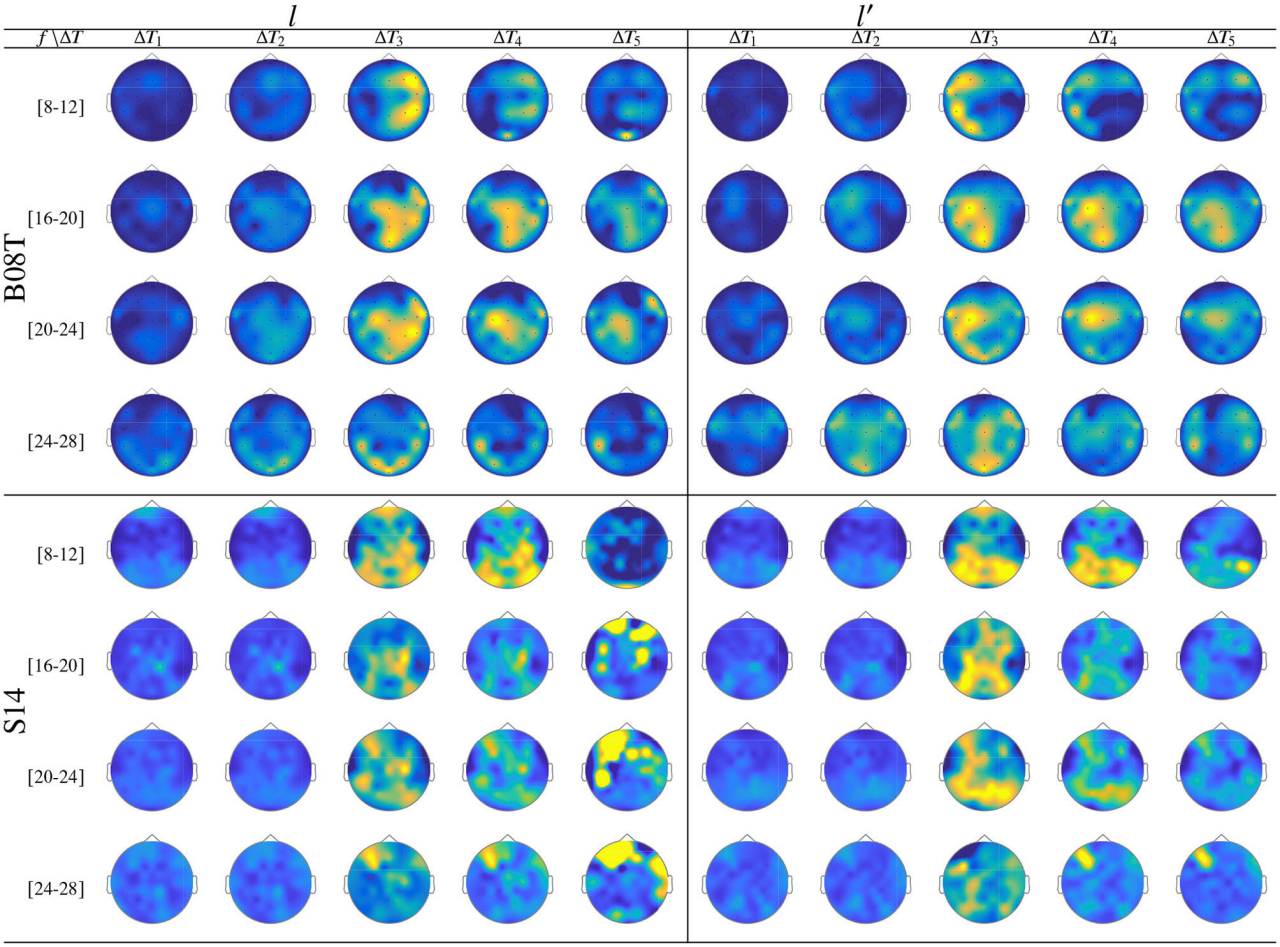
ERD/S analysis. Topoplots of the extracted dynamics **θ**_ζ_(*f*, τ; *c*|λ), showing the dominance to different extents, performed by *B08T* (class *l* left side) and (class *l*′ right side).

As seen in Figure 9, while there is no neural activity measured before the cue Δ*T*_1_ regardless of the frequency band and performed tasks. The main dynamics take place over the interval Δ*T*_3_, showing a higher contribution of MI-related bands (namely, [8−−12], [16−−20], and [20−−24] Hz) as reported for hand movement tasks (Pfurtscheller et al., 2000). Afterward, the ERDS-based dynamics decrease over time (Δ*T*_4_ and Δ*T*_5_). Note the asymmetrical contribution of the contralateral electrodes for each label.

### 3.5. Results of Multi-Subject Dynamic Models

Finally, we compute the collective task-related dynamics extracted from the *t-f* feature patterns $\left\{\theta_{\eta }^{\left(m\right)}\left(f,\tau |\lambda \right):\forall f\in \Omega ,\tau \in N_{\tau }\right\}$ with η = {*J*, ζ, ϕ} using a time-window length, fixed for each extraction method differently. Namely, τ_*J*_ = 1*s*, τ_ϕ_ = 0.1*s*, and τ_ζ_ = 0.004*s*, resulting in the following volumes of time samples: *J* → *N*_τ_ = 60, ϕ → *N*_τ_ = 66, ζ → *N*_τ_ = 1751. Then, we assess the similarity of each accomplished model of collective dynamics with the corresponding subject-level dynamics. However, for interpretability purposes, the similarity measure is computed just over the primary motor area as the most representative in motor imagery tasks (Neuper and Pfurtscheller, 2001).

**Figure 10:**
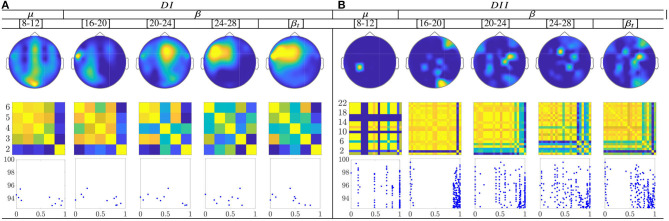
Common neural dynamics of Rayleigh Quotient, estimated over the subject set within the MI interval Δ*T*_3_. **(a)** Topographic *t-f* representation of multi-subject model. **(b)** Pairwise distances estimated by desegregating individuals from the multi-subject model. **(c)** Scatter plot of normalized distances values of assessed group dynamics.

The topographic representation in Figure 10 shows that the CSP-based multi-subject model does not vary remarkably along with μ and β oscillations, within the MI interval Δ*T*_3_. Although the RQ relation resumes the influence of both labels into a single value, the neural activation is reflected over the primary motor and parietal areas, which should be strongly activated in MI tasks.

In the case of the Rayleigh Quotient, the topographic representation shows that the multi-subject model of extracted CSP dynamics for dataset D-I changes remarkably along with *μ* and *β* oscillations, within the MI interval ΔT_3_, as seen in **Figure 12**. Also, the neural activation is reflected in the primary motor and parietal areas, which should be strongly activated in MI tasks. For the database D-II, the contribution is placed in the sensorimotor area too, but the *β* oscillation influences the most.

**Figure 11:**
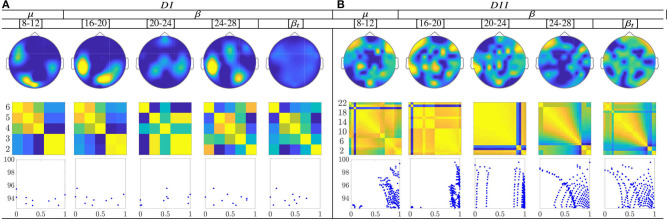
Common neural dynamics of Functional connectivity, estimated over the subject set within the MI interval Δ*T*_3_. **(a)** Topographic *t-f* representation of multi-subject model. **(b)** Pairwise distances estimated by desegregating individuals from the multi-subject model. **(c)** Scatter plot of normalized distances values of assessed group dynamics.

The calculated topograms of the common functional connectivity dynamics (see top row in Figure 11) reveal perceptible differences between tasks. Figure 11 (second row) shows the influence of stepwise removing the subjects with lower accuracy from the performed group analysis. As observed, the multi-subject model of DI (see the second row) changes significantly, meaning that the RQ time-series does not preserve enough the observed relationship between the subject-level dynamics (see the bandwidths [16−−20] and β). This finding can be better understood in the third row that displays the scatter plots of the performed similarity measure estimated for the resulting groups. Several reasons may account for this result: the low number of subjects, the low resolution of EEG montage, and the high heterogeneity between their dynamics, as previously reported. In the case of DII, the procedure of subject's removal reveals that their influence gathers into several groups depending on the spectral bandwidth.

**Figure 12:**
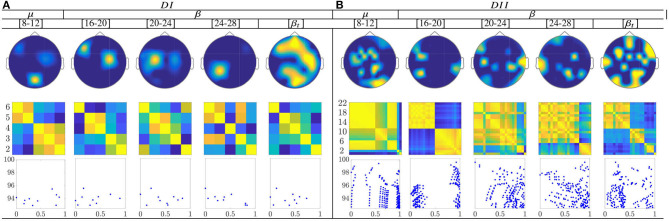
Common neural dynamics of Event-related de/synchronization, estimated over the subject set within the MI interval Δ*T*_3_. **(a)** Topographic *t-f* representation of multi-subject model. **(b)** Pairwise distances estimated by desegregating individuals from the multi-subject model. **(c)** Scatter plot of normalized similarities values of assessed group dynamics.

For the multi-subject models of ERD/S-based dynamics, the topograms of both datasets in Figure 12 show a relevant contribution that is located in the primary motor area, supplementary motor cortex, and parietal cortex. These facts may have a physiological interpretation related to MI practice. Thus, channels present a notable neural activity through the considered frequencies, excluding the highest bandwidth [20−−24] and [24−−28] Hz.

Extracted from the ERD/S-based dynamics, the applied inter-subject similarity measure allows identifying the presence of subgroups very accurately, having a close resemblance between their produced neural connectivity patterns (see second-row of Figure 12). Thus, the subjects with the highest accuracy gather the first subdivision, while the individuals with lower accuracy are the last instance. Moreover, the scatter plots (third row) make evident of subgroups for the bandwidths [8−−12], [16−−20], and [20−−24] Hz for bot datasets. Therefore, the multi-subject performed by ERD/S-based dynamics are effective in reducing subject aggregation. Thus, the group-level model preserves the main properties of similarity, even after removing subjects with lower accuracy in discriminating between MI tasks, although the efficiency depends on the frequency bandwidth.

## 4. Discussion and Concluding Remarks

Here, we develop a dynamic model for estimating the common neural activity across subjects to provide new insights into the evolution of collective mental imagery processes. After the preprocessing stage, the *t-f* EEG signal set is fed into a feature extraction algorithm to improve the efficiency of triggering activity representation. Then, we employ a statistical thresholding algorithm to extract a multi-subject model that provides a set of confident estimates contributing the most to discriminating between MI tasks. We compare three feature extraction methods for making group inferences from subject-level dynamic information of neural activity. The obtained validation results indicate that the estimated collective dynamics reflect the flow of sensorimotor cortex activation differently. Therefore, the common model addresses inter-subject and inter-trial variability sources individually, depending on the engaged extraction method.

The developed group dynamic model can be considered a valid and promising approach to infer the main properties of multi-subject datasets; however, the following remarks should be highlighted:

–*Feature extraction of neural dynamics*. To build up the subject-level feature sets, a common representational space, **θ**(*f*, τ|λ), is proposed that encodes the electrode (spatial) contribution, evolving through time and frequency domains. To address sources of inter-subject and inter-trial variability of individuals, a *t-f* feature set is extracted, for which the domain parameters (time window length and filter bandwidth setup) are selected to be the more relevant in discriminating between MI tasks, yielding a distinct dimensionality of each extracted characteristic set. Because of the difference in the captured dynamics, each engaged extraction method differently reflects the flow of sensorimotor cortex activation during the analyzed representative MI intervals.
Three feature extraction methods were compared, providing insight into the possible limitations. Namely, the CSP algorithm that encodes the joint influence of both labels reflects the neural activation over the areas related to MI tasks. However, it demands a very wide time window, decreasing the accuracy of the performed *t-f* feature dynamics so that the variability of inter-subject dynamics increases notably for higher bandwidths. The Functional connectivity method allows differentiating between both spatial patterns of MI activity, though the background neural activity notably changes the inter-subject dynamics. Lastly, the ERD/S patterns enable representing more accurately the evolution of MI paradigms, including the subject dominance of the contralateral electrodes for each task.
A significant concern is the reproducibility of FC together with the derived graph measures, which tend to worsen as the scalp montage size increases. In particular, there is a negative correlation between the inter-electrode distance and inter-electrode wPLI, making this estimator ineffective to detecting zero-lag phase differences, as discussed by Hardmeier et al. (2014).
–*Group analysis*. The multi-subject model enables inferring collective task-related dynamics from extracted subject-level feature sets. For better interpret the results, we evaluate the effectiveness of gathering data from collective sources by introducing two assumptions: (i) a non-linear assessment of the similarity between multi-subject data originating subject-level dynamics, instead of the widely used correlation index, as in Velásquez-Mart́ınez et al. (2019). (ii) an assessment of brain network development though the ranking of subject accuracy in performing the MI task classification. As a result, the performed dynamic common model proves the ability to preserve the spatial distribution of brain neural activity throughout time and spectral domains, obtained from each one of the single-subject models. The attained multi-subject model allows spatial patterns that accommodate essential individual differences in sources.
However, some issues affect the ability to collect latent structures from sources. The employed collective framework extracts the latent components consistently expressed in the population, implying that they perform under the same conditions. In practice, this premise seems to be far from being right. Thus, several subjects systematically complete the MI tasks in the wrong way, misleading the group analysis. Hence, due to differences in individual MI literacy, the intra-subject heterogeneity tends to considerably reduce the estimated multi-subject models. To illustrate, the presence of ERD/S mechanisms activated at the ipsilateral electrode in several subjects results in incorrect estimated values of hemisphere contribution. Thus, the subject triad performing the worst (probably, with modest motor imagery abilities) should be segregated in a different group.
Besides, the employed latent component decomposition is unsupervised, and one might be interested in extracting the most discriminating dynamics to avoid the influence of some background neural activity. One more concern is the raised computational burden related to the *t-f* dynamic modeling, reducing to a small number of analyzed subjects.

As future work, the authors plan to involve more effective stochastic approaches for representing domain-evolving dynamics, including feature extraction methods based on Gaussian processes or Markov models. Also, the use of supervised analysis will enhance the interpretation of assessed multi-subject models. We also intend to carry out the validation of databases with a higher population.

## Data Availability Statement

Publicly available datasets were analyzed in this study. These data can be found here: http://www.bbci.de/competition/iv/ and http://gigadb.org/dataset/100295.

## Author Contributions

GC-D and LV-M conceived and designed the idea of the present work. LV-M and FZ-C organized and pre-processed the EEG data involved in all computational procedures. GC-D and LV-M verified the analytical methods, supervised the findings of this work, and contributed to their interpretation. All authors discussed the results and contributed to the final manuscript.

## Conflict of Interest

The authors declare that the research was conducted in the absence of any commercial or financial relationships that could be construed as a potential conflict of interest.

## Footnotes

^1^BCI Competition IV, publicly available at: www.bbci.de/competition/iv/.

^2^http://gigadb.org/dataset/100295.

^3^http://biosig.sourceforge.net.
